# The Growth Trend Predictions in Pulmonary Ground Glass Nodules Based on Radiomic CT Features

**DOI:** 10.3389/fonc.2020.580809

**Published:** 2020-10-20

**Authors:** Chen Gao, Jing Yan, Yifan Luo, Linyu Wu, Peipei Pang, Ping Xiang, Maosheng Xu

**Affiliations:** ^1^Department of Radiology, The First Affiliated Hospital of Zhejiang Chinese Medical University (Zhejiang Provincial Hospital of Traditional Chinese Medicine), Hangzhou, China; ^2^The First Clinical Medical College of Zhejiang Chinese Medical University, Hangzhou, China; ^3^GE Healthcare Life Sciences, Hangzhou, China

**Keywords:** machine learning, growth, solitary pulmonary nodule, tomography, X-ray computed, nomograms

## Abstract

**Background:** The management of ground glass nodules (GGNs) remains a distinctive challenge. This study is aimed at comparing the predictive growth trends of radiomic features against current clinical features for the evaluation of GGNs.

**Methods:** A total of 110 GGNs in 85 patients were included in this retrospective study, in which follow up occurred over a span ≥2 years. A total of 396 radiomic features were manually segmented by radiologists and quantitatively analyzed using an Analysis Kit software. After feature selection, three models were developed to predict the growth of GGNs. The performance of all three models was evaluated by a receiver operating characteristic (ROC) curve. The best performing model was also assessed by calibration and clinical utility.

**Results:** After using a stepwise multivariate logistic regression analysis and dimensionality reduction, the diameter and five specific radiomic features were included in the clinical model and the radiomic model. The rad-score [odds ratio (OR) = 5.130; *P* < 0.01] and diameter (OR = 1.087; *P* < 0.05) were both considered as predictive indicators for the growth of GGNs. Meanwhile, the area under the ROC curve of the combined model reached 0.801. The high degree of fitting and favorable clinical utility was detected using the calibration curve with the Hosmer-Lemeshow test and the decision curve analysis was utilized for the nomogram.

**Conclusions:** A combined model using the current clinical features alongside the radiomic features can serve as a powerful tool to assist clinicians in guiding the management of GGNs.

## Introduction

The detection rate of pulmonary nodules has been significantly increased since the introduction of low dose CT screening, especially for the ground glass nodule (GGN) (1, 2). The GGN, which includes pure and part-solid GGN, is defined as a hazy region of increased opacity on lung windows without obscurity to bronchial and vascular structures (3). The pathophysiology of GGN is based on the accumulation of fluid, cells or amorphous material in the alveoli itself, or thickening of the alveolar walls and septal interstitium (4). The GGN is observed in many lesions, such as malignant tumors and benign lesions which include inflammatory lesions, interstitial lung disease, and so on (5–7). In 2017, the Fleischner society released new guidelines for GGN management, with more aggressive guidelines toward follow up (8). Although radiologists were able to observe changes of GGN in follow-up CT examinations, most GGNs progress at a slow rate, particularly persistent GGN (9). Therefore, long windows of follow-up are often required. This is a source of great anxiety for patients and their families. Furthermore, the increased duration of follow up often increases the rate of no-shows. Therefore, several studies have sought to provide a greater diagnostic indicator for the growth of GGNs through the analysis of traditional imaging features, such as diameter and CT attenuation (10–13). For example, Matsuguma et al. showed there were significant differences in diameter between rapidly growing and non-growing pure GGNs (12). However, distinguishing the growing GGNs from static GGNs using traditional quantitative CT imaging remains a distinctive challenge.

With the advancements in imaging technology, many radiologists attach importance to these parameters, such as the Gray-Level Co-occurrence Matrix (GLCM) and the Gray Level Run-length Matrix (RLM) (14). While there have been many radiomic pulmonary studies in recent years, there have been no studies comparing and contrasting radiomic features with clinical features to predict the growth of GGNs (15, 16).

Therefore, the purpose of the current study is to compare the performance of clinical signatures and radiomic features in predicting the growth of GGNs and to build a clinical-radiomic nomogram to accurately predict the growth of GGNs.

## Materials and Methods

### Patients

This retrospective study was approved by our institutional review board and informed consent requirement was waived.

Between September 2012 and December 2018, a total of 85 patients with 110 GGNs were involved in this study which included 68 patients with a single GGN, 11 patients with two GGNs, 5 patients with three GGNs, and 1 patient with five GGNs. The inclusion criteria were as follows: (1) detected pulmonary nodules showed GGNs on non-enhanced CT thin-sectioned images; (2) the GGN diameter between 5 and 30 mm in the initial CT image; (3) there were more than two follow-up, thin-section CT examinations and the follow-up interval was longer than 2 years.

The exclusion criteria were as follows: (1) biopsy, radiotherapy, chemotherapy or surgical resection during any follow-up; (2) severe respiratory artifacts on CT images; (3) a history of lung surgery; (4) the first or final CT examinations were low dose CT examinations.

**Figure 1:**
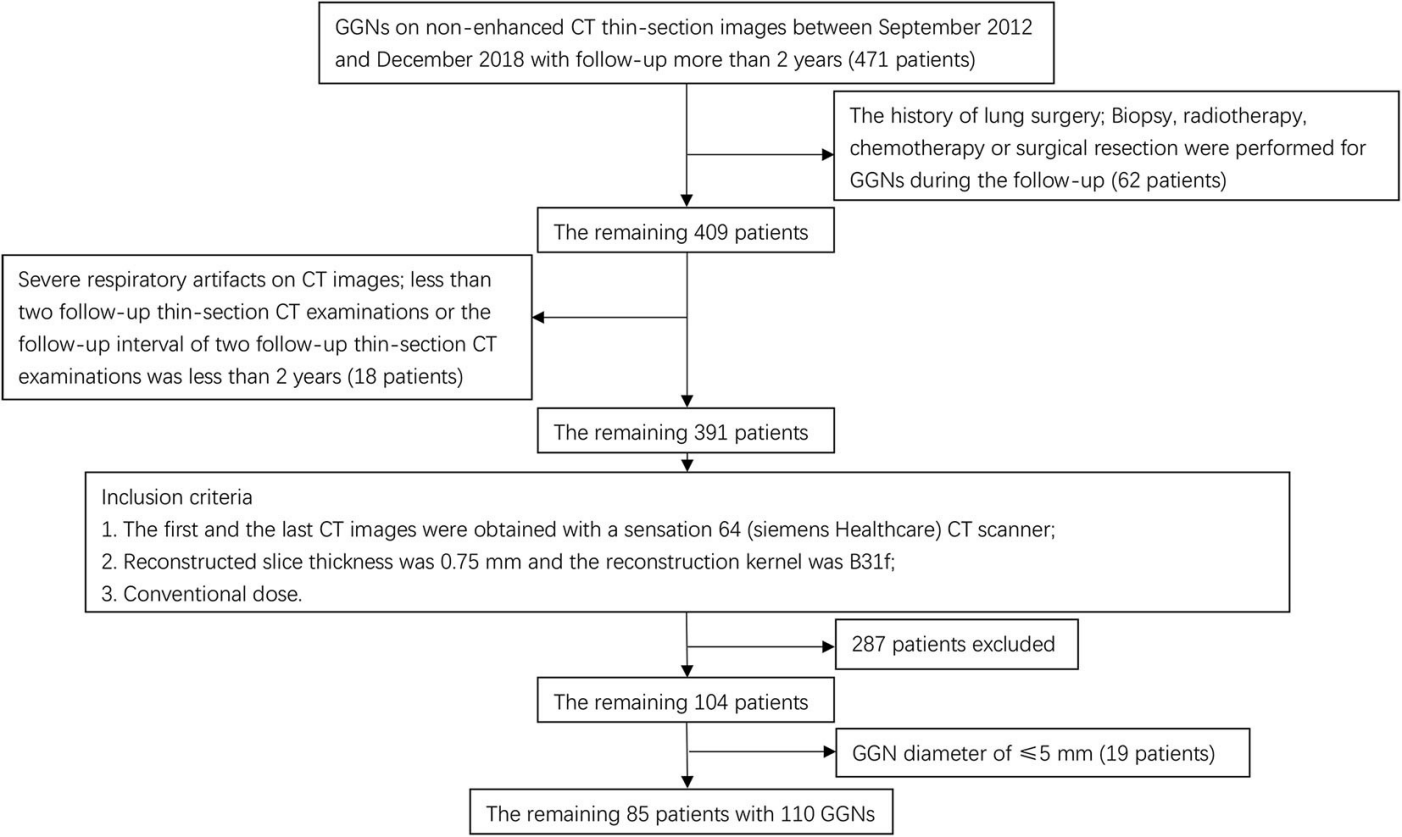
Flow diagram of GGN selection.

The patients were randomly divided into a training cohort and a validation cohort. A flow chart of patients who were selected is presented in Figure 1.

### CT Examination Acquisition

All images were obtained with a Siemens Somatom sensation, 64 slice, CT scanner (Siemens Healthcare). The imaging parameters were as follows: tube voltage, 120 kVp; automatic tube current modulation; collimation, 0.6 mm^*^64; matrix, 512^*^512; rotation time, 0.37 s, reconstruction slice thickness, 0.75 mm with a 0.5 mm interval, reconstruction kernel, B31f.

### Nodule Selection and Growth Definition

The GGNs were assessed by two radiologists (one with 11 years of experience and another with 4 years of experience in pulmonary radiology) based on thin-section unenhanced CT images. All GGNs were confirmed by two radiologists with consensus agreement. If the two radiologists cannot reach a consensus, the GGNs were assessed by a third-party professor with 28 years of experience in pulmonary radiology. The diameter of GGN was defined as the maximum length of on the transverse lung window in thin-section CT images. The solid diameter was defined as the maximum size of the solid portion of GGNs on the transverse lung window in thin-section CT images. All measurements in the initial and final CT images were constructed from transverse sections by two radiologists to reach a consensus. To eliminate measuring error, growth was defined as an increase in diameter or the size of the solid portion ≥2 mm, or an emerging solid portion (17).

### Region of Interest Segmentation and Feature Extraction

All regions of interest (ROI) were manually segmentation by a radiologist with 4 years of experience in pulmonary radiology on initial thin-section CT images by using ITK-SNAP 3.6.0 (www.itksnap.org), and further verified by another radiologist with 11 years of experience in pulmonary radiology. For situations of a discrepancy between the two radiologists, the segmentation patterns were evaluated by a professor of radiology with 28 years of experience in pulmonary radiology. Normal structures within or around the GGNs, such as vessels and pleura, were not included in ROIs. A total of 396 radiomic features were quantitatively extracted using Analysis Kit software (AK, GE Healthcare). These features included single-order (histograms and morphologic features) alongside higher-order parameters (Supplementary Descriptions). The higher-order parameters were described as “texture” features, such as GLCM and RLM. Texture features described statistical interrelationships between voxels with similar (or dissimilar) contrast values. The values of each feature for all GGNs were normalized with Z-scores ((x – μ)/σ) for the purpose of removing the unit limits of each feature before being applied to a machine learning model for classification. For the model parameters, x represents the value of the feature, μ indicates the average feature values in all GGNs within the cohort, and σ represents the corresponding standard deviation.

### Radiomic Feature Selection and Construction of Rad-Score

The minimum Redundancy Maximum Relevance (mRMR) and the least absolute shrinkage and selection operator (LASSO) algorithm were used to select radiomic features (18). Initially, mRMR was performed to eliminate redundant and irrelevant features. The top 20 features were selected, and the optimized subset of features was chosen by LASSO to construct the final model. After the number of features was determined, the most predictive feature subset was chosen and the corresponding coefficients were calculated. Rad-score was composed by summing the chosen features, weighted by their coefficients, and comparing it between the training group and test group. The performance of the model was then evaluated by ROC analysis.

### Establishment of the Clinical and Combined Model

In the establishment of the clinical model, a univariate logistic regression was used to evaluate the clinicopathological factors. Factors with a *P*-value < 0.05 were considered in the stepwise multivariate logistic regression analysis. Meanwhile, the Akaike information criterion (AIC) was used to determine a stopping rule. A combination of the clinical signatures from the clinical model and rad-score were used to develop the combined model with multivariate logistic regression. Afterward a test process was implemented.

### Model Comparison and Nomogram Establishment

The predictive accuracy of the three models was assessed by the area under the curve (AUC) in both the training and validation group. The AIC of the clinical model was applied to identify the most appropriate clinical model. The probability of growth for each GGN was analyzed by logistic regression, and GGNs were grouped into growth and non-growth cohorts based on the highest Youden index. According to the actual growth results, the accuracy, specificity, sensitivity, negative-predictive value (NPV) and positive-predictive value (PPV) for the three models were calculated in the training and test group. Then, the nomogram of the most appropriate model was established. According to the reference of Iasonos et al. and Stephenson et al., the usefulness of a nomogram is that it maps the predicted probabilities into points on a scale from 0 to 100 in a picture and the total points accumulated by the various factors correspond to the predicted probability for a patient (19, 20). Meanwhile, the calibration curves measured the consistency between the actual growth probability and the predicted growth probability to evaluate the running characteristic of the nomogram. The degree of fit of the prediction models was also evaluated by the Hosmer-Lemeshow (H-L) test.

### Development of Decision Curve Analysis (DCA)

To assess the added value of radiomic features to clinical in the prediction of the growth of GGNs, three DCA was performed based on clinical diagnosis, radiomics, and the combined model. The clinical application of said model could be verified by quantifying the net benefits for a range of threshold probabilities.

### Statistical Analysis

R statistical software (version 3.5.1) was used for all statistical tests. A chi-square test or Fisher's exact test was used for the categorical variable. A student's *t*-test, Mann-Whitney *U*-test or Kruskal-Wallis *H*-test was used for the continuous variable. The “mRMRe” package was used to perform the mRMR model, and the “glmnet” package was used to conduct the LASSO model. The “pROC” package was used to plot the ROC curves and the “rms” package was used to build nomogram and perform calibration curves. The ROC curve analysis was performed using the “ROC.TEST” packages. Meanwhiles, the “generalhoslem” package and the “dca.R.” package were used to conduct the H-L test and DCA, respectively. A two-sided *P* < 0.05 was considered statistically significant.

## Results

### Clinical Characteristics and Development of the Clinical Model

The baseline of GGNs is shown in Tables 1, 2. There were no significant differences between the training and validation cohorts (Table 1). The difference in clinical characteristics between non-growth group and growth group was shown in Table 2. The univariate logistic regression analysis demonstrated the type, diameter, solid-diameter, and volume as risk signatures for the growth of GGNs. However, only the diameter was still considered as a viable predictive indicator in the clinical model after using the stepwise multivariate logistic regression analysis as shown in Table 3.

**Table 1:** Characteristics of the GGNs in the training and validation group.

**Variable**	**Sample**	**Training cohort**	**Validation cohort**	***P*-value**
Gender, No. (%)				1.000
Male	32	23 (29.5%)	9 (28.1%)	
Female	78	55 (70.5%)	23 (71.9%)	
Location, No. (%)				0.163
RUL	56	34 (43.6%)	22 (68.8%)	
RML	7	6 (7.7%)	1 (3.1%)	
RLL	13	10 (12.8%)	3 (9.4%)	
LUL	23	18 (23.1%)	5 (15.6%)	
LLL	11	10 (12.8%)	1 (3.1%)	
Type, No. (%)				0.863
Pure GGN	83	58 (74.4%)	25 (78.1%)	
Part-solid GGN	27	20 (25.6%)	7 (21.9%)	
Age	110	56.8 ± 11.9	59.2 ± 16.2	0.403
Diameter	110	8.1 ± 3.8	8.5 ± 2.9	0.580
Soliddiameter	110	0.9 ± 1.9	1.0 ± 2.7	0.846
MeanCT	110	−700.2 ± 84.7	−706.2 ± 91.2	0.739
StdCT	110	103.5 ± 35.0	96.5 ± 39.9	0.366
Volume	110	418.0 ± 539.1	431.1 ± 335.3	0.898

P-value is derived from statistical analyses between each of variables and two cohort.

Continuous variables are expressed as the mean ± standard deviation and categorical variables are expressed as the number. A chi-square test or Fisher's exact test was used for the categorical variable. A student's t-test, Mann-Whitney U-test or Kruskal-Wallis H-test was used for the continuous variable. All statistical analyses for the present study were performed with R (version 3.5.1). A two-tailed p-value < 0.05 indicated statistical significance.

*GGN, ground glass nodule; LLL, left lower lobe; LUL, left upper lobe; RLL, right lower lobe; RML, right middle lobe; RUL, right upper lobe*.

**Table 2:** Characteristics of the non-growth and growth cohorts.

**Characteristics**	**Training set (*****n*** **=** **78)**			**Validation set (*****n*** **=** **32)**		
	**Non-growth (*n* = 52)**	**Growth (*n* = 26)**	***P***	**Non-growth (*n* = 22)**	**Growth (*n* = 10)**	***P***
**Gender**						
Male	14 (26.9%)	9 (34.6%)	0.661	6 (27.3%)	3 (30.0%)	1.000
Female	38 (73.1%)	17 (65.4%)		16 (72.7%)	7 (70.0%)	
**Age (years)**	56.8 ± 11.2	57.0 ± 13.3	0.947	55.5 ± 17.3	67.3 ± 10.0	0.044
**Location**			0.151			0.565
RUL	27 (51.9%)	7 (26.9%)		15 (68.2%)	7 (70.0%)	
RML	2 (3.8%)	4 (15.4%)		1 (4.5%)	0 (0.0%)	
RLL	7 (13.5%)	3 (11.5%)		2 (9.1%)	1 (10.0%)	
LUL	10 (19.2%)	8 (30.8%)		4 (18.2%)	1 (10.0%)	
LLL	6 (11.5%)	4 (15.4%)		0 (0.00%)	1 (10.0%)	
**Nodule type**			0.035			0.033
Pure GGN	43 (82.7%)	15 (57.7%)		20 (90.9%)	5 (50.0%)	
Part-solid GGN	9 (17.3%)	11 (42.3%)		2 (9.1%)	5 (50.0%)	
**Diameter**	6.8 ± 1.7	10.7 ± 5.3	<0.001	7.9 ± 2.1	9.9 ± 3.9	0.063
**Solid diameter**	0.5 ± 1.1	1.8 ± 2.7	0.002	0.3 ± 0.9	2.6 ± 4.4	0.015
**Mean CT**	−710.3 ± 82.1	−680.0 ± 88.0	0.133	−710.5 ± 93.1	−696.9 ± 90.9	0.701
**StdCT**	98.0 ± 30.8	114.4 ± 40.7	0.046	89.0 ± 32.3	113.1 ± 50.9	0.103
**Volume**	248.3 ± 252.2	757.3 ± 765.3	<0.001	337.5 ± 284.7	637.1 ± 360.1	0.011

P-value is derived from statistical analyses between each of variables and two cohort.

Continuous variables are expressed as the mean ± standard deviation and categorical variables are expressed as the number. A chi-square test or Fisher's exact test was used for the categorical variable. A student's t-test, Mann-Whitney U-test or Kruskal-Wallis H-test was used for the continuous variable. All statistical analyses for the present study were performed with R (version 3.5.1). A two-tailed p-value < 0.05 indicated statistical significance.

GGN, ground glass nodule; LLL, left lower lobe; LUL, left upper lobe; RLL, right lower lobe; RML, right middle lobe; RUL, right upper lobe.

**Table 3:** Stepwise multivariate logistic regression analysis.

**Variable**	**AIC**
Type + Diameter + Solid diameter + StdCT + Volume	91.078
Type + Diameter + Solid diameter + StdCT	89.103
Diameter + Solid diameter + StdCT	87.122
Diameter + StdCT	85.131
Diameter	83.696

*AIC, Akaike information criterion*.

### Features Selection and Construction of Model

**Figure 2:**
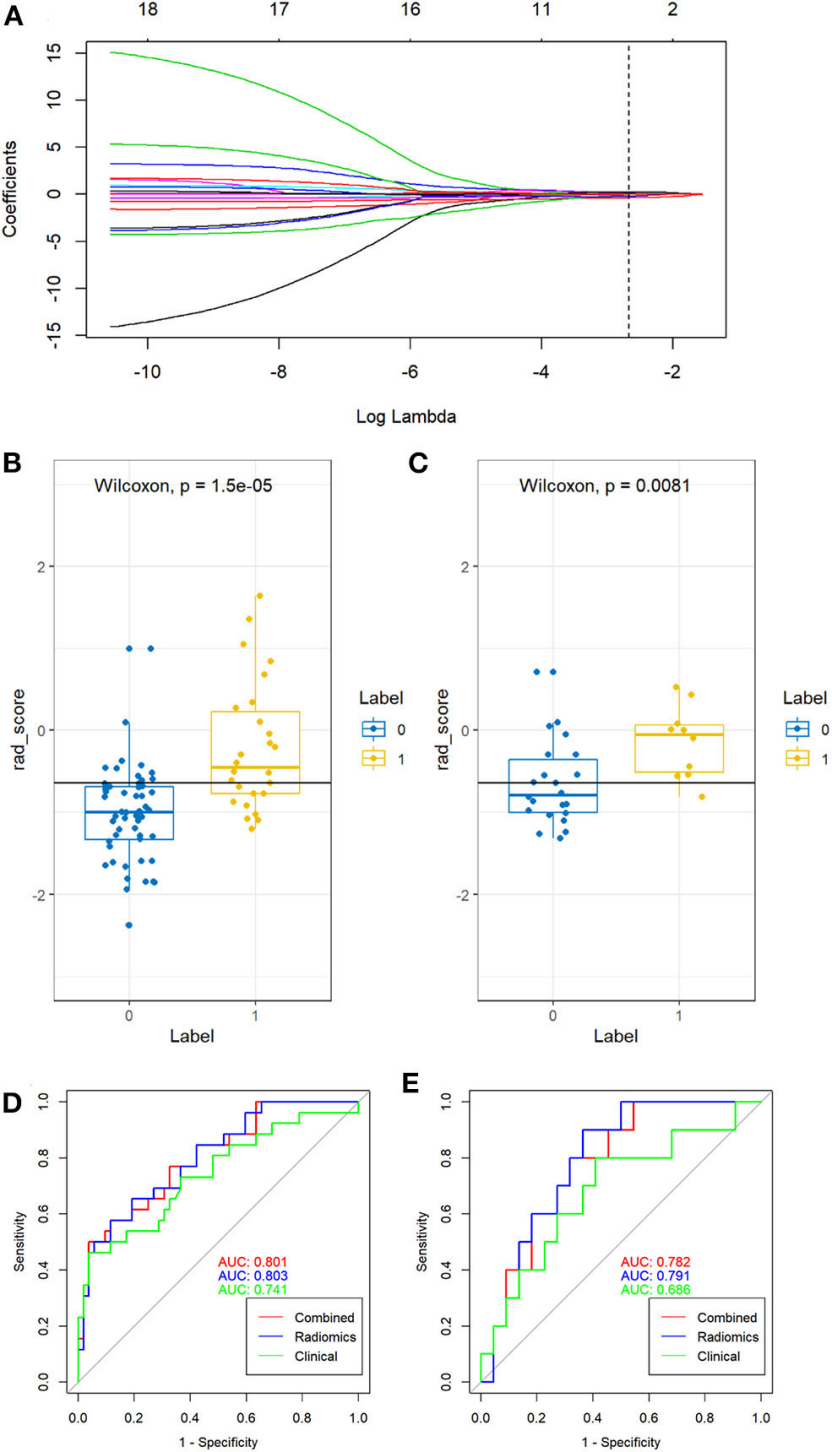
Feature selection and the performance of rad-score and three models. **(A)** The least absolute shrinkage and selection operator (LASSO) regression was used to choose the features to construct the final model. **(B,C)** The rad-score from non-growth (class 0) and growth (class 1) on the training group **(B)** and test group **(C)** were compared, respectively. **(D,E)** Receiver operating characteristic (ROC) curve for predicting the growth of GGNs in the training group **(D)** and test group **(E)**.

By using the mRMR method, 20 features were retained. Then, five features after regression by the LASSO model. These features were presented in the rad-score and were calculated by using the following formula: rad-score = 0.088^*^RunLengthNonuniformity_AllDirection_offset7_SD-0.367^*^SurfaceVolumeRatio-0.214^*^LongRunLowGreyLevelEmphasis_angle0_offset1+0.03^*^ShortRunEmphasis_AllDirection_offset1_SD+0.227^*^VolumeCC-0.753. As seen in Figure 2, rad-score was significantly different between the growth and non-growth groups in both the primary and test cohort (*P* < 0.01) when using the Wilcoxon rank-sum test. The rad-score [odds ratio (OR) = 5.130; 95%CI: 0.948–37.835; *P* < 0.01] and diameter (OR = 1.087; 95%CI: 0.785–1.564; *P* < 0.05) were both considered as predictive indicators for the growth of GGNs by using the multivariate logistic regression analysis as seen in Table 4. In the combined model, the rad-score was the key predictive indicator of the growth of GGNs.

**Table 4:** Risk factors for the growth of GGNs.

**Variable**	**Univariate logistic regression**		**Multivariate logistic regression**	
	**OR (95% CI)**	***P*-value**	**OR (95% CI)**	***P*-value**
Gender	0.696 (0.253–1.957)	0.483	NA	NA
Age	1.001 (0.962–1.043)	0.946	NA	NA
Location	1.279 (0.940–1.758)	0.121	NA	NA
Type	3.504 (1.225–10.377)	0.020	NA	NA
Diameter	1.427 (1.189–1.832)	0.001	1.087 (0.785–1.564)	0.047
Solid diameter	1.505 (1.137–2.107)	0.009	NA	NA
Mean CT	1.004 (0.999–1.010)	0.141	NA	NA
StdCT	1.014 (1.000–1.029)	0.055	NA	NA
Volume	1.003 (1.001–1.005)	0.006	NA	NA
Rad-score	7.438 (2.866–26.466)	<0.001	5.130 (0.948–37.835)	0.001

*CI, confidence interval; GGN, ground glass nodule; NA, not available; OR, odds ratio*.

### Model Comparison and Construction of the Nomogram

**Figure 3:**
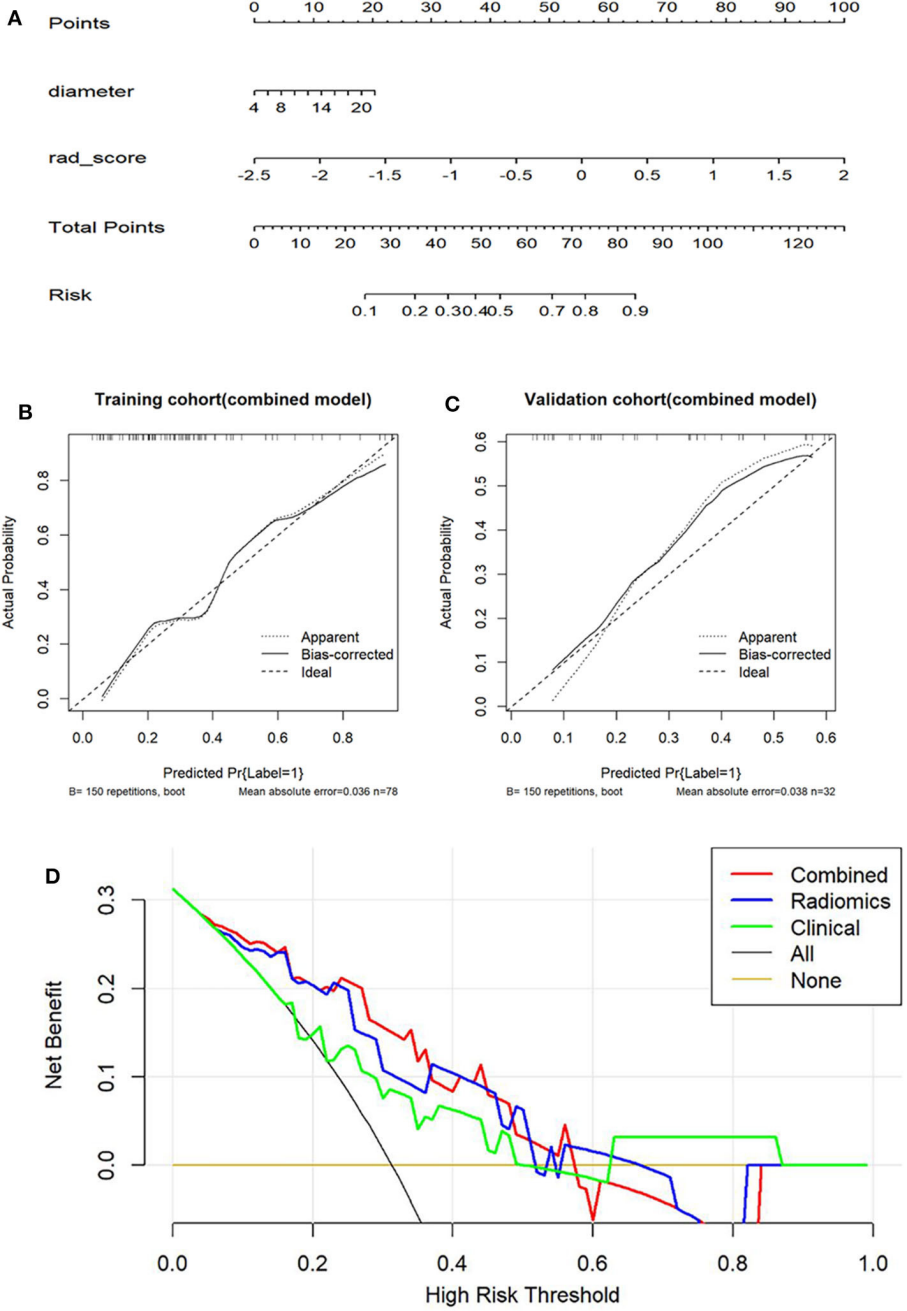
The evaluation of the degree of fitting for the combined model and comparison of clinical utility of three models. **(A)** The combined nomogram based on clinical factors and rad-score for predicting the growth of GGNs. **(B,C)** Calibration curves for prediction of the growth of GGNs based on the combined model in two cohorts. The X-axis represents the predicted probability of GGN growth based on the combined model and the Y-axis is the actual probability for the growth of GGNs. **(D)** The X-axis represents high-risk threshold and the Y-axis represents net benefit. The green line represents the clinical model. The blue and red line, respectively represent the radiomic model and the combined model. The black line represents a hypothetical GGN growth. The yellow line a non-growing GGN.

For the training group, the AUC of the combined model, the radiomic model, and the clinical model were 0.801, 0.803, and 0.741, respectively. For the validation cohort, the AUC of the combined model, the radiomic model, and the clinical model were 0.782, 0.791, and 0.686, respectively. There were significant differences between the ROC of the combined model and clinical model (*Z* = 1.987, *P* = 0.047). No significant differences were found between the ROC of the combined model and radiomics model (*Z* = −0.490, *P* = 0.624). Meanwhile, the combined model also showed the greatest accuracy (accuracy: 80.8%; sensitivity: 86.7%; specificity: 79.4%; PPV: 50.0%; NPV: 96.2%) in the prediction of the growth of GGNs (Table 5). Therefore, the nomogram was generated based on the combined model (Figure 3). Compared to the diameter, the rad-score made up a high proportion of the nomogram.

**Table 5:** Accuracy and predictive value between three models.

	**AUC**	**95% CI**	**Sensitivity**	**Specificity**	**Accuracy**	**PPV**	**NPV**
**TRAINING COHORT**							
Clinical model	0.741	0.617–0.866	0.857	0.781	0.795	0.462	0.962
Radiomics model	0.803	0.700–0.905	0.808	0.654	0.756	0.824	0.630
Combined model	0.801	0.698–0.904	0.867	0.794	0.808	0.500	0.962
**VALIDATION COHORT**							
Clinical model	0.686	0.475–0.897	0.500	0.750	0.688	0.400	0.818
Radiomics model	0.791	0.635–0.947	0.636	0.900	0.719	0.933	0.529
Combined model	0.782	0.620–0.944	0.533	0.882	0.719	0.800	0.682

AUC, area under the curve; CI, confidence interval; NPV, negative-predictive value; PPV, positive-predictive value.

As the calibration plots illustrates, there is a high degree of consistency between actual observation and the combined model in both the training and the validation cohort (Figure 3). Meanwhile, the results of the H-L test were non-significant statistics in the training group (*P* = 0.6305) and test group (*P* = 0.6698), which represented a good fitting model.

### Clinical Use of DCA

The DCA based on three models was shown in Figure 3. However, the DCA based on the radiomic model showed a greater benefit in the prediction of GGN growth in the 10–60% threshold probabilities as opposed to the clinical model. In essence, the diagnostic utility of the rad-score surpassed that of the clinical model within this threshold range. Combining the clinical features and rad-score allowed for a DCA similar to that of the radiomic model.

## Discussion

In this study, the predictive value of radiomic features was analyzed to determine their utility in predicting the growth of GGNs. For the combined model, an improvement in diagnostic utility was also evident through the rad-score for predicting the growth of GGNs. The combined model showed the performance with an AUC of 0.801. Then, the results of DCA showed that the combined model and the radiomic model demonstrated greater performance over the clinical model in predicting the growth of GGNs (Figure 3).

Before this study, the majority of investigations used conventional CT features to predict the growth of GGNs. Parameters, such as the diameter, CT attenuation, volume, and shape were commonly used (21, 22). For example, Masaya Tamura et al. retrospectively analyzed the potential value of conventional CT features including diameter, and mean CT attenuation in the prediction of the growth of GGNs (22). Their result showed the mean CT attenuation (OR = 7.572; *P* = 0.0023) was the best predictor of the growth of GGNs by using multivariate analysis. However, in our study, the diameter of GGNs was found to be significant indicator of GGN growth in the clinical model after using a stepwise multivariate logistic regression analysis. A potential explanation is that conventional CT features require visual inspection and measurement on a macro scale. In essence, features less than the resolution of the equipment are missed. CT radiomic features offer the advantage of extracting high-throughput data from CT images (23). Therefore, many studies using radiomic methods to analyze GGNs have been reported (24–26). A retrospective study by Li Fan et al. analyzed the value of radiomic features as a potential biomarker for the distinction between pre-invasive lesions and invasive lung adenocarcinoma appearing as GGNs (25). A separate study by Q. Sun et al. sought to investigate the relationship between CT texture features and the growth trends of GGNs (27). Features quantitatively extracted from 89 GGNs including the mean value, uniformity, entropy, and energy, were used in their analysis. Their results showed that there was a significant relationship between uniformity and volume doubling time for pure GGNs (*P* = 0.022). In our study, the radiomic model were compared alongside the clinical and combined model for the evaluation of GGNs. The SurfaceVolumeRatio and VolumeCC were considered key parameters for the accuracy of the proposed model according to its corresponding coefficients. Specifically, the SurfaceVolumeRatio was defined as the surface area of lesions in square millimeters divided by the volume of lesions in cubic centimeters using AK. The SurfaceVolumeRatio was inversely related to the rad-score using corresponding coefficients, which suggested that the value of SurfaceVolumeRatio may be large in non-growth GGNs. According to the general pathophysiology, the SurfaceVolumeRatio is relatively small in irregular GGN which may indicate heterogeneity and malignancy (28). Therefore, the SurfaceVolumeRatio is inversely related to the degree of malignancy, further confirming the findings made herein. According to definitions of these radiomic features (Supplementary Descriptions), the RunLengthNonuniformity, LongRunLowGreyLevelEmphasis, and ShortRunEmphasis also reflect the heterogeneity of lesions. The definition of VolumeCC was set as the volume of GGNs in cubic centimeters using AK. Based on the corresponding coefficients, the value of VolumeCC was positively correlated with the rad-score in our study, suggesting that the value of VolumeCC may be larger in the growth group. Results by Jacob Scharcanski et al. indicated that the growth pattern of pulmonary nodules is exponential (29). Therefore, GGNs with large volumes will have a faster growth rate over smaller nodules. These findings are in line with those obtained herein. Additionally, the mRMR method was used to select radiomic features, which can then evaluate the correlation between the radiomic features and results and the correlation between different radiomic features (30). The aim was to select the features most relevant to the results and remove redundant features. In the current study, the top 20 features selected from 396 total features were used in the mRMR model. By doing so, the accuracy of feature selection was greatly increased. Once the nomogram was developed, a calibration curve and H-L test were performed to evaluate the predictive model (31). The results of both studies suggested that the combined model showed a good correlation with the actual data.

Despite the findings presented herein, several limitations are of note. Firstly, the design of the retrospective study meant that sample sizes were small as a result of the strict inclusion criteria. There was also a lack of external validation as data was gathered from a single institution. Secondly, the growth of GGNs was only measured in two dimensions. Yet, a volume change may better reflect the growth of GGNs as their growth can often be in asymmetric axis (32). Several studies have also investigated the use of volume double time and mass double time to reflect the growth rate of GGNs (33, 34). Yet, current controversy exists as to the best method to measure the natural history of GGNs. The use of manual segmentation in the current study also predisposes results to observer bias. Furthermore, clinical confounding variables, such as patients' social and family history were not included in this analysis, as information regarding such variables is insufficient in the current long-term study. Specifically, the smoking history of patients was not elicited, despite smoking history is a potential confounding factor in the growth of GGNs (35–37). Finally, pathological data from GGNs were not obtained, meaning that one cannot infer as to the relationship between the results presented and the types of GGNs with their respective growth trends.

In conclusion, this study has developed and compared three models for predicting the growth of GGNs; the current clinical model, the radiomic model and a combination of the two. The results suggested that the radiomic model and the combined model showed increased utility in predicting the growth of GGNs as opposed to the clinical model. The nomogram studies conducted herein suggested the combined model as offering the greatest diagnostic value. Therefore, this study indicates the utility and versatility of the combined model in guiding the management of GGNs.

## Data Availability Statement

The raw data supporting the conclusions of this article will be made available by the authors, without undue reservation.

## Ethics Statement

The studies involving human participants were reviewed and approved by the Ethics Committee of the First Affiliated Hospital of Zhejiang Chinese Medical University. Written informed consent for participation was not required for this study in accordance with the national legislation and the institutional requirements.

## Author Contributions

CG: investigation, formal analysis, data curation, and writing–original draft. JY, YL, and LW: investigation and visualization. PP: software, validation, formal analysis, and visualization. PX: investigation. MX: conceptualization, methodology, resources, writing–reviewing and editing, supervision, and project administration. All authors contributed to the article and approved the submitted version.

## Conflict of Interest

PP was employed by GE Healthcare Life Sciences. The remaining authors declare that the research was conducted in the absence of any commercial or financial relationships that could be construed as a potential conflict of interest.

## Abbreviations

AIC: akaike information criterion
AK: Analysis Kit
AUC: area under the curve
CI: confidence interval
CT: computed tomography
DCA: decision curve analysis
GGNs: ground glass nodules
GLCM: gray-level co-occurrence matrix
H-L: Hosmer-Lemeshow
LASSO: least absolute shrinkage and selection operator
LLL: left lower lobe
LUL: left upper lobe
MRMR: minimum redundancy maximum relevance
NA: not available
NPV: negative-predictive value
OR: odds ratio
PPV: positive-predictive value
RLL: right lower lobe
RLM: gray level run-length matrix
RML: right middle lobe
ROC: receiver operating characteristic
ROI: regions of interest
RUL: right upper lobe.
