# Supplementary material for: The Growth Trend Predictions in Pulmonary Ground Glass Nodules Based on Radiomic CT Features
Source: Front Oncol. 2020 Oct 20;10:580809. doi: 10.3389/fonc.2020.580809 (PMC7606974; doi:10.3389/fonc.2020.580809)
Supplement: Supplementary file 1 [file Table_1.DOCX]

**Supplementary Material**

**Supplementary Descriptions**

Radiomic Features Extraction

A total of 396 radiomic features were quantitatively extracted using AK software (Analysis Kit, GE Healthcare, US). These features included single-order (histograms and morphologic features) alongside higher-order parameters. The following text was used to define these significant features.

The volume (𝑉) of the tumor was determined by counting the number of pixels in the tumor region and multiplying this value by the voxel size.

The surface area (A) was calculated by triangulation (i.e. dividing the surface into connected triangles) and was defined as:

$$A=\sum_{i=1}^{N} \frac{1}{2}|a_{i}b_{i}\times a_{i}c_{i}|$$

Where 𝑁 was the total number of triangles covering the surface and 𝑎, 𝑏 and 𝑐 were edge vectors of the triangles.

The SurfaceVolumeRatio was defined as the surface area of lesions in square millimeters divided by the volume of lesions in cubic centimeters.

$$SurfaceVolumeRatio=\frac{A}{V}$$

The grey level run-length matrix (RLM) 𝐏𝐫 (𝐢, 𝐣 | 𝛉) was defined as the numbers of runs with pixels of gray level *i* and run length *j* for a given direction θ. RLMs were generated for each sample image segment having directions (0°,45°,90° &135°).

1. Short Run Emphasis (SRE)

$$SRE(\theta)=\frac{1}{n_{r}}\sum_{i=1}^{M} \sum_{J=1}^{N} \frac{p(i,j,\theta)}{j^{2}}$$

2. Long Run Low Gray-Level Emphasis (LRLGE)

$$LRLGE(\theta)=\frac{1}{n_{r}}\sum_{j=i}^{N} \sum_{i=1}^{M} \frac{p(i,j,\theta)\cdot j^{2}}{i^{2}}$$

3. Run Length Nonuniformity (RLN)

$$RLN(\theta)=\frac{1}{n_{r}}\sum_{j=i}^{N} {(\sum_{i=1}^{M} p(i,j,\theta))}^{2}$$
